# Emerging pathways of communication between the heart and non-cardiac organs

**DOI:** 10.7555/JBR.32.20170137

**Published:** 2018-03-28

**Authors:** Eugenio Hardy-Rando, Carlos Fernandez-Patron

**Affiliations:** 1. Biotechnology Laboratory, Study Center for Research and Biological Evaluations, Institute of Pharmacy and Foods, University of Havana, Havana PO Box 430, Cuba; 2. Department of Biochemistry, Cardiovascular Research Centre, Mazankowski Alberta Heart Institute, Faculty of Medicine and Dentistry, University of Alberta, Edmonton, AB T6G 2H7, Canada.

**Keywords:** heart, liver, metabolism, inflammation, natriuretic peptides, microRNA, matrix metalloproteinase

## Abstract

The breakthrough discovery of cardiac natriuretic peptides provided the first direct demonstration of the connection between the heart and the kidneys for the maintenance of sodium and volume homeostasis in health and disease. Yet, little is still known about how the heart and other organs cross-talk. Here, we review three physiological mechanisms of communication linking the heart to other organs through: i) cardiac natriuretic peptides, ii) the microRNA-208a/mediator complex subunit-13 axis and iii) the matrix metalloproteinase-2 (MMP-2)/C-C motif chemokine ligand-7/cardiac secreted phospholipase A2 (sPLA2) axis – a pathway which likely applies to the many cytokines, which are cleaved and regulated by MMP-2. We also suggest experimental strategies to answer still open questions on the latter pathway. In short, we review evidence showing how the cardiac secretome influences the metabolic and inflammatory status of non-cardiac organs as well as the heart.

## Introduction

Diseases with strong metabolic and inflammatory components (ischemic heart disease, arthritis, neurodegeneration and cancer) are leading causes of morbidity, extremely high costs associated with health care, and mortality worldwide^[1]^. Given the incomplete understanding of these diseases, new studies to decipher organ-specific cross-talk between inflammation and metabolic pathways are constantly needed.

This paper aims to: (i) introduce historical background for the groundbreaking notion that the heart exerts an endocrine function^[2^–^5]^, (ii) review briefly two other recent mechanisms- the cardiac-specific microRNA (miR)-208a/mediator complex subunit-13 (MED13) axis^[6]^ and the matrix metalloproteinase 2 (MMP-2)/C-C motif chemokine ligand 7 (CCL7)/cardiac secretory phospholipase A2 (sPLA2) axis- by which the heart modulates lipid metabolism in non-cardiac organs (e.g., the liver)^[7^–^9]^ and (iii) suggest experimental approaches to elucidate the mechanism that regulates the cardiac-specific origin of sPLA2 in MMP-2 deficiency as well as delineate the biochemical pathway by which the dyad of MMP-2 and monocyte-chemotactic protein 3 (MCP-3)/CCL7 modulates cholesterol homeostasis in the heart and other organs. Outputs of this latter line of research have the potential to open up new avenues for modulating cholesterol metabolism [e.g., at the levels of MMP-2 activity or signaling mediators positioned downstream of C-C chemokine receptor type 2 (CCR2)] in inflammatory and metabolic disorders.

## The heart has an endocrine function consisting of the release of natriuretic peptides

Rows 1 to 8 in *Table 1* show selected discoveries related to the ability of the heart atrial muscle cells of mammals to synthesize, store within specific atrial granules, and release cardiac natriuretic peptides (cNPs). The mechanism of action of cNPs is summarized in *Box 1*. These findings by the research team of Dr. de Bold were the first direct demonstration that the heart has an endocrine function (summarized in *Table 1*, rows 1 to 8). In *Table 2*, we summarize selected observations describing how the cNP system influences hypertrophic growth, fibrosis, and cardiac remodeling/dysfunction. For a summary of the influences of cNPs on adipose tissue biology and metabolism, please see *Table 3.*

**Tab.1:** Discovery of cardiac natriuretic peptides and the heart-specific miR-208a/MED13 axis

Molecules investigated; Research question; Authors	Author’s main results and conclusions
ANP; What are the acute renal effects of the extract of rat atrial myocardium? (de Bold AJ *et al*., 1981)^[2]^	The atrial extract (i) decreases blood pressure and slightly increases hematocrits; (ii) rapidly increases the concentration and urinary excretion of sodium and chloride (≥30-fold), the urine volume (~10-fold), the potassium excretion (two-fold); (iii) contains a potent natriuretic and chloriuretic factor, which strongly inhibits the renal tubular NaCI reabsorption.
ANP; Can extracts from other sources induce a natriuretic and diuretic response? (de Bold AJ and Salerno TA, 1983)^[3]^	Natriuresis and diuresis is induced by atrial extracts from all mammalian species, frog atrial and ventricular extracts, hen ventricle extracts (only diuresis), and not by hen atrial extracts or rat tissue extracts other than the atrial extract; (ii) Natriuretic activity is restricted to heart.
ANP; What are the molecules responsible for these activities? (de Bold AJ and Flynn TG, 1983)^[4]^	Cardionatrin I, which also has effect on vascular smooth muscle tone, has a molecular mass of 5.1 kDa by urea-SDS-PAGE and 49 amino acid residues one of which is cysteine.
ANP; What is the common precursor of Cardionatrin I and other atrial peptides? (Flynn TG *et al*., 1985)^[5]^	(i) Cardionatrin IV, consisting of 126 amino acids, has a molecular mass of 19 kDa by urea-SDS-PAGE, and begins immediately after the signal peptide sequence of procardionatrin at residue 25. It does not contain residues 151 and 152, which are arginines; (ii) Cardionatrin III begins at residue 73 and Cardionatrin I begins at residue 123; (iii) Cardionatrins I, III, many cleavage fragments thereof and numerous versions of the carboxyl terminal portion of Cardionatrin I are products derived from a common precursor, Cardionatrin IV; (iv) Cardionatrins I-IV peptides are derived from preprocardionatrin, a common precursor of 152 amino acids (in the rat); (v) The biologically active sequences of the atrial natriuretic factor is contained in the COOH-terminal portion of the molecule.
BNP; Identification in porcine brain of a novel natriuretic peptide (Sudoh T* et al*., 1988)^[10]^	BNP contains 26 amino acid residues, two Cys residues, seven amino acid substitutions and one addition of (Arg) compared to α-ANP; (ii) BNP possesses diuretic-natriuretic (e.g., increase in urine output, Na^+^, K^+^, Cl^−^ excretion) and hypotensive (decrease in mean blood pressure) responses similar to that of ANP; (iii) There may be a dual mechanism involving both ANP and BNP to control physiological functions such as water intake and salt appetite.
BNP; What is the intracellular localization of BNP in human cardiac myocytes? (Nakamura S *et al*., 1991)^[11]^	BNP is specifically localized in only some of the secretory granules of the human atrium and ventricle that contain ANP, as shown with different patients (with aortic regurgitation, mitral regurgitation or autoptic); (ii) The atrium is the major production site of BNP; (iii) Together, ANP and BNP allows the human heart to regulate blood pressure and body fluid.
ANP and BNP; Natriuretic peptides circulate in blood (Clerico *et al.*, 2011 and citations therein)^[12]^	(i) Posttranslational processing of proBNP is required for secretion and bioactivity- this process is impaired in patients with heart failure leading to biologically inactive BNP; (ii) proBNP-derived fragments (e.g., the intact and glycosylated forms of precursor proBNP, NH2-terminal-truncated BNP form 3-32) circulate in human plasma in addition to bioactive BNP1-32; (iii) In plasma of patients with heart failure, a significant portion of immunoreactive B-type related peptides is comprised of intact or glycosylated forms of proBNP- this suggests that plasma proteases cleave the circulating proBNP to produce biologically active BNP; (iv) In experimental models and in patients with chronic heart failure, a resistance to ANP and BNP is observed; possible mechanisms of resistance to biological effects of ANP and BNP may operate at: a) the pre-receptor level (e.g., existence of inactive natriuretic peptides in plasma, increase in inactivation and degradation of active natriuretic peptides, decreased renal filtration), b) receptor level (e.g., downregulation of NPR-A and NPR-B in target tissues, altered ANP/BNP receptor binding or desensitization, or c) post-receptor level (e.g., altered intracellular signaling).
CNP; Are cardiomyocytes able to produce CNP? (Del Ry S *et al.*, 2011)^[13]^	(i) Both HUVEC and H9c2 muscle cells express CNP (150 and 200 bp); which can be confirmed in neonatal rat primary cardiomyocytes; (ii) CNP can be immunodetected in both H9c2 cells (by radioimmunologic assay) and cardiomyocytes of pig hearts; (iii) CNP is constitutively expressed in cardiomyocytes.
ANP and BNP; Biological factors and pathophysiological mechanisms that stimulate the production/release of natriuretic peptides (Clerico *et al*., 2011 and citations therein)^[12]^	(i) The production/release of cNPs is stimulated by: a) Ang II, ET1, α-adrenergic agonists, cytokines such as IL-1, IL-6 and TNF-α, and lipopolysaccharide (all of which signal throughout NF-kB activated by MAPK), b) arginine vasopressin (through Ca_2_^+^ influx and PKC), c) GFs (signaling through MAPK cascade), d) prostaglandins (through PLC, IP3, PKC, and MLCK), e) chromogranin B (through NF-kB and IP3/ Ca_2_^+^ influx), f) thyroid hormones (through thyroid hormone regulatory element), g) corticosteroids (through glucocorticoid responsive element), and h) estrogens; (ii) The production and release of BNP from ventricular cardiomyocytes is stimulated by inflammation, ventricular hypertrophy, and fibrosis; (iii) Even in isolated and cultured ventricular cells, myocardial ischemia can induce the synthesis/secretion of BNP and its related peptides; (iv) Both ANP and BNP transcription may be activated by the hypoxia-inducible factor-1a (which is induced under low oxygen conditions).
Cardiac specific miR-208a and MED13; How does cardiac MED13 influence whole body metabolism? (Grueter CE *et al*., 2012)^[6]^	(i) Pharmacologic inhibition of the cardiac-specific miR-208a confers resistance to diet-induced obesity (e.g., smaller visceral WAT and subscapular BAT, normal glucose response, lower fasting insulin levels) with beneficial metabolic effects (e.g., reduced serum triglyceride and cholesterol levels); (ii) miR-208a is a negative transcriptional regulator of MED13 in the heart. Among the functions of MED13 are: (a) to inhibit expression of metabolic genes regulated by NRs (e.g., Gpd2, Thrsp, Cidea, Elovl6, Eno1, PPARγ Tkt), (b) to control whole-body metabolic homeostasis (e.g., αMHC-Med13 TG mice, with increased cardiac expression of MED13, show enhanced metabolic rate, diminished serum triglyceride and cholesterol levels, resistance to diet-induced obesity including less fat mass versus WT littermates, reduced visceral WAT and subscapular BAT mass as well as less adipocyte size and less lipid accumulation, improved glucose (tolerance) response, lowered plasma lipid levels, and improved whole-body insulin sensitivity, and (c) regulate energy expenditure (e.g., increased oxygen consumption, carbon dioxide production) in mice; (iii) MED13 deficiency in the heart increases susceptibility to metabolic syndrome and diet-induced obesity in mice, as shown with Med13 cardiac knockout mice versus Med13fl/fl littermates on HF diet. (iv) Circulating factors may relay MED13 activity from the heart to other organs but these factors remain elusive.

Abbreviations: miR-208a: microRNA-208a; MED13: mediator complex subunit 13; ANP: atrial natriuretic peptide; SDS-PAGE: sodium dodecyl sulfate-polyacrylamide gel electrophoresis; BNP: brain natriuretic peptide; proBNP: ventricular circulating inactive precursor of BNP; CNP: C-type natriuretic peptide; HUVEC: human umbilical vein endothelial cell; NRs: nuclear receptors; TG: transgenic; Gpd2: glycerol-3-phosphate dehydrogenase 2; Thrsp: thyroid hormone responsive; Cidea: cell death-inducing DFFA-like effector A; Elovl6: ELOVL fatty acid elongase 6; Eno1: Enolase 1; PPARγ: peroxisome proliferator activated receptor gamma; Tkt: Transketolase; WT: wild-type; WAT: white adipose tissue; BAT: brown adipose tissue; HF: high fat; Ang II: angiotensin II; ET1: endothelin-1; NF-kB: nuclear factor kappa-light-chain-enhancer of activated B cells; MAPK: p38 mitogen-activated protein kinase; PKC: protein kinase C; GFs: growth factors; MLCK: myosin light chain kinase; PLC: phospholipase C.

**Box 1:** On the biology of cardiac natriuretic peptides

As illustrated with ANP in the scheme, ANP and BNP are synthesized as preprohormones, stored as prohormones in secretory granules, processed into mature forms, and continuously secreted from the heart[^14^]; CNP is also constitutively synthesized within the heart[^13^]. Picomolar concentrations are found in the plasma of healthy subjects: ANP (3.2–19.5 pmol/l), BNP (1.4–14.5 pmol/L), CNP (1–6 pmol/L)[^15^–^18^]. The main release mechanism is exocytosis of vesicles budding from immature atrial granules[^19^–^20^]. Release is stimulated by mechanical stretch of atrial muscle, change in hemodynamic load, sympathetic stimulation and a variety of agonists and pathophysiological mechanisms (*Table 1*, row 9). Natriuretic peptide receptor (NPR)-A, NPR-B and NPR-C mediate effects of cardiac natriuretic peptides. Binding to NPR-A and NPR-B, which are guanylyl cyclase-coupled receptors, catalyzes the conversion of guanosine-5'-triphosphate (GTP) to cyclic guanosine monophosphate (cGMP)[^21^]. Elevated levels of cGMP elicit various biological actions through different effectors (e.g., cGMP-gated ion channels, cGMP-dependent protein kinases, cGMP-regulated cyclic nucleotide phosphodiesterases)[^22^]. Cardiac natriuretic peptides impact natriuresis, diuresis, improve glomerular filtration rate, suppress the renin-angiotensin-aldosterone system, inhibit plasma renin activity, induce systemic vasodilation, and arterial hypotension. Target systems include heart, arteries, kidney, brain, liver, gut[^23^–^39^] as well as adipose tissue (thus also enhancing lipolysis), skeletal muscle (where they increase oxidative capacity and mitochondrial biogenesis), and pancreas (thus improving insulin secretion). Thus, cardiac natriuretic peptides link the functions of the heart and other non-cardiac organs[^40^–^43^]. LBD: ligand binding domain; KHD: kinase homology domain; HR: hinge region; GCD: guanylyl cyclase domain; PKG: protein kinase G; PDE: phosphodiesterase; CNG: cyclic nucleotide-gated ion channels; cAMP: cyclic AMP; IP3: inositol trisphosphate; ERK: extracellular-signal-regulated kinase; IL-6: interleukin 6.	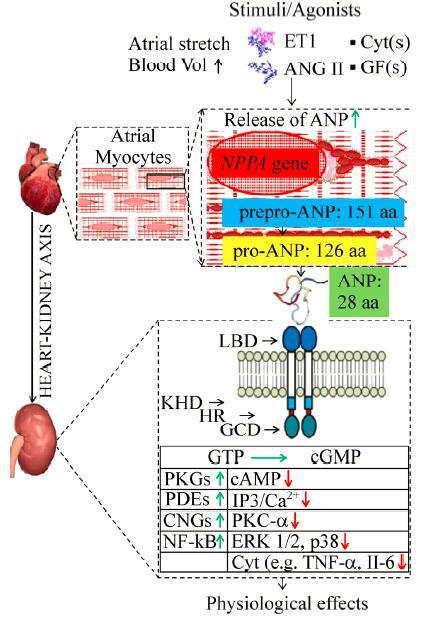

**Tab.3:** Mechanisms linking the cardiac natriuretic system and heart issues and involving MMPs/cytokines

Question	Selected observations
The role of ANP in cardiac hypertrophy and remodeling (Wang *et al.*, 2005)[^44^]	The mechanism by which ANP protects against cardiac hypertrophy induced by pressure overload involves the negative regulation of genes encoding MMP-2 in mice subjected to transverse aortic constriction [e.g., 2-fold increase in *Nppa*^+/+^ and 3-fold increase in *Nppa*^-/-^] and TIMP-3 as well as other protein factors related to extra cellular matrix deposition (e.g., collagen I/III, osteopontin, periostin, thrombospondin).
The role of NPRA/cGMP signaling in regulation of MMPs and other factors (e.g., proinflammatory mediators) (Vellaichamy *et al.*, 2005)[^45^]	(*i*) There is a link between *Npr1* gene disruption in mice and the expression and activation of matrix metalloproteinases (e.g., MMP-2, MMP-9) and pro-inflammatory cytokines that play critical roles in cardiac hypertrophy, fibrosis, and extra cellular matrix remodeling; (*ii*) Hearts from *Npr1*^-/-^homozygous mice at an early age versus age-matched WT (*Npr1*^+/+^) control mouse hearts show strongly activated genes (e.g., MMP-2 and MMP-9 by 3-5-fold, TNF-α by 8-fold) - these genes remain activated in adult mice; (*iii*) In *Npr1*^-/-^ mice treated with GM6001, a MMP inhibitor, the activities of MMP-9 and MMP-2 are decreased (between 3 and 5 fold), fibrosis is reduced (75%), ventricular dilatation is attenuated, and fractional shortening is improved. These observations implicate MMPs in myocardial fibrosis and cardiac hypertrophy.
The effect of *Npr1* gene copy numbers on the expression of cardiac hypertrophic and fibrotic markers, proinflammatory mediators, and MMPs (Subramanian *et al.*, 2016)[^46^]	(*i*) *Npr1* gene-disrupted heterozygous (*Npr1*^-/-^, 1-copy) mice versus WT (*Npr1*^-/-^, 2-copy) mice and gene duplicated (*Npr1*^-/-^, 3-copy) mice, show an augmented heart weight to body weight ratio, elevated blood pressure, downregulated expression of TIMP-1 (by 36%) and TIMP-2 (by 40%) mRNA transcripts, and increased hypertrophic markers, proto-oncogenes, NF-kB, and MMPs (MMP-2 [e.g., 2.59-fold], MMP-9 [e.g., 1.93-fold]) - this leads to fibrosis and hypertrophic remodeling. (*ii*) Retinoic acid and butyrate block histone deacetylases and activate histone acetyltransferases to activate Npr1 gene transcription. In *Npr1*^-/-^ (1-copy) mice treated with all-trans retinoic acid or sodium butyrate the expression of NF-kB and MMP-2/MMP-9 is reduced, the expression of TIMP-1 and TIMP-2 is upregulated, fractional shortening is increased, systolic and diastolic parameters of the *Npr1*^-/-^ hearts are decreased; (iii) In age-matched 2- and 3-copy cardiac tissues, the expression of fibrotic markers is strongly reduced and the expression of TIMPs activated, after drug treatment (with retinoic acid, sodium butyrate or in combination).

Abbreviations: *Nppa*: pro-ANP gene; TIMP: tissue inhibitor of metalloproteinase; *Npr1*: NPR-A gene.

**Tab.4:** Effects of the cardiac natriuretic peptide system in the cardio-adipose axis

Question	Selected observations
The role of the cardiac natriuretic peptide system in adipose tissue biology and metabolism (Sarzani *et al.*, 2008 and citations therein)[^47^]	(i) NPR-C is the second largest expressed receptor in adipose tissue; (ii) NPR-C expression in adipose tissue is strongly and selectively downregulated by fasting; (iii) Higher clearance and/or diminished activity of cNPs in the subcutaneous adipose tissue of obese hypertensive patients; (iv) Decrease effectiveness of ANP in obese premenopausal women; (v) Increased effects (cGMP, natriuresis, diuresis, blood pressure) of ANP on obese hypertensive patients after a very-low-calorie diet; (vi) In obese hypertensive subjects, a variant of the promoter of the *NPR-C* gene is linked to: a) higher blood pressure, b) lower ANP levels, c) augmented abdominal circumference, and d) the risk of developing abdominal obesity in male; (vii) ANP inhibits the proliferation of human visceral differentiated pre-adipocytes and mature adipocytes; (viii) Natriuretic peptides are strong lipolytic hormones, a property that is enhanced by diet and weight loss; (ix) Natriuretic peptides regulate the supply of non-esterified fatty acids to the working muscles (both cardiac and skeletal); (x) An improved metabolism in the heart and muscles would result from the net effect of natriuretic peptides on adipose and muscle tissues; (xi) The inhibitory effect induced by ANP-induced lipolysis is counteracted by ANP-stimulated secretion of adiponectin; (xii) The secretion of cytokines such as IL-6 and TNF-α and several chemokines as well as adipokines (leptin, retinol-binding protein-4) involved in inflammation and insulin resistance is inhibited by ANP; (xiii) The biological activity (local and systemic) of natriuretic peptides is increased by downregulation of NPR-C due to fasting and calorie restriction; (xiv) Natriuretic peptides could behave as anti-obesity peptides in the presence of uncoupling activity involving non-esterified fatty acids and mitochondrial burning for ATP synthesis; (xv) Ang II may hamper the positive effects of natriuretic peptides; (xvi) In obese subjects with and without hypertension or heart failure, circulating levels of natriuretic peptides and NH2-terminal-proANP and NH2-terminal-proBNP are reduced; (xvii) Reduced NH2 terminal-proBNP levels correlate with hepatic steatosis and higher hematocrit values in elderly patients.
Pathophysiological links between obesity (visceral fat distribution) and cardiac endocrine function (low plasma BNP levels) (Clerico *et al.*, 2012 and citations therein)[^48^]	(i) There is an inverse correlation between BMI (e.g.,≥30 kg/m2) and plasma BNP and NH2-terminal-proBNP values, both in healthy subjects and in patients with heart failure; (ii) The increased expression of NPR-C in the adipose tissue may lead to the peripheral clearance of BNP; nevertheless, this increase in NPR-C should have no influence in modulating the NH2-terminal-proBNP levels in obese subjects. (iii) Some alterations of peripheral degradation of ANP and BNP in individuals having the corin I555 (P568) allele; (iv) The production and secretion of BNP may be regulated by the gonadal function including the estrogens/androgens circulating ratio; (v) The activity of cardiac endocrine function is depressed by the presence of one or more circulating or tissue bioactive substances (e.g., leptin); (vi) An efficacious (standard) pharmacological treatment of obese individuals or patients with hypertension and/or type-2 diabetes mellitus tends to decrease significantly the production/secretion of BNP and NT-proBNP; (vii) The inverse correlation between BMI and BNP values in patients with heart failure (e.g., with hemodynamic impairment, activation of counteracting neurohormone, increased circulating levels of cytokines) may be partially explained by the effect of the malnutrition-inflammation complex syndrome.
Effects of natriuretic peptide system in obesity, metabolic syndrome and type 2 diabetes (Moro, 2016 and citations therein)[^49^]	(i) Natriuretic peptides promotes fat mobilization and utilization as well as regulate energy metabolism and expenditure; (ii) In obesity, metabolic syndrome and type 2 diabetes there is a state of natriuretic peptide system deficiency (natriuretic handicap); (iii) Contributing factors to the natriuretic handicap are the elevated levels of glucose, insulin, fatty acids, pro-inflammatory cytokines; (iv) The natriuretic handicap occurs despite a higher left-ventricular mass and end-diastolic pressure in people with obesity compared to lean individuals; (v) Human and mouse adipose tissue express NPR-C; high fat feeding up-regulates NPR-C and reduces NPRA mRNA – as confirmed in studies with mice; (vi) Obese diabetic db/db mice show: a) white fat, brown fat and skeletal muscles containing strongly up-regulated NPR-C protein levels, b) down-regulated NPRA protein levels, and c) a strong decrease of plasma BNP levels; (vii) Mice with diet-induced obesity show reduced NPRA expression (mRNA and protein); (viii) Insulin up-regulates NPR-C expression in adipocytes; (ix) ANP infusion in healthy volunteers increases adiponectin levels; (x) Natriuretic peptides induce the expression/secretion of adiponectin in cultured human adipocytes; (xi) In healthy Japanese men, serum NH2 terminal-proBNP is inversely associated with metabolic risk and positively with adiponectin; (xii) Systemic insulin sensitivity is enhanced by adiponectin and reduced by IL-6 and TNF-α secretion from adipose tissue; (xiii) Humans volunteers show a blood glucose-lowering effect of infused BNP to an oral glucose challenge; (xiv) In humans, whole-body insulin sensitivity correlates (positively) with NPR-A expression in adipose tissue and skeletal muscle; (xv) In adipocytes of obese individuals with and without type 2 diabetes, NPR-A is down-regulated and NPR-C is up-regulated (mRNA and protein); (xvi) Natriuretic peptides protect human skeletal muscle against palmitate-induced lipotoxicity and insulin resistance; (xvii) A robust link between natriuretic peptide signaling and type 2 diabetes is demonstrated by studies with transgenic mice of the natriuretic peptide system.
The link between endocrine function, adipose tissue, and sex steroid hormones (Clerico *et al.*, 2011 and citations therein)[^12^]	(i) Adipokines (e.g., leptin) can differently regulate the production/secretion of ANP and BNP by cardiomyocytes throughout multiple metabolic pathways; these pathways are activated or inhibited depending on different pathophysiological conditions; (ii) A cross talk exits between the cardiac endocrine function and adipose tissue (e.g., ANP and BNP regulate fat tissue function and growth as well as exerting potent lipolytic effects in human fat cells, ANP increases production of adiponectin); (iii) ANP gene expression is increased in a dose-dependent manner by female sex steroids; (iv) Estradiol and progesterone are required for normal ANP gene expression by rat cardiomyocytes; (v) In postmenopausal women, a stimulatory action on the production/secretion of ANP and BNP is induced by hormone replacement therapy with female steroid hormones; (vi) There is debate on the effects of androgens on the production/secretion of ANP and BNP (e.g., in atrial cultured myocytes of newborn rats, the synthesis and secretion of both ventricular and atrial ANP is stimulated by testosterone; whereas in castrated male rats, the concentration of ANP in plasma and ANP atrial stores is increased; testosterone replacement reduces ANP levels in plasma, but not in ANP atrial stores; blocking the androgen receptor and, to a lesser extent, suppressing androgen in men with prostate cancer, increases NH2-terminal-proBNP levels in plasma; other studies [e.g., the Dallas Heart Study] in humans suggest that estrogens may stimulate whereas androgens inhibit the production/secretion of ANP and BNP).

Abbreviations: ATP: adenosine 5’-triphosphate; BMI: body mass index.

## A cardiac-specific miR-208a/MED13 axis further connects the heart with other organs

Recent studies^[6^,^50^–^51]^ have identified cardiac-specific miR-208a as a negative regulator of the subunit 13 of mediator complex MED13 (summarized in *Table 1*, row 10). MED13 regulates the transcription of many nuclear receptor genes involved in fatty acid oxidation as well as influencing the activity of as-yet-unidentified secreted/circulating factors, which connect the activity of cardiac MED13 with the metabolism and energy homeostasis program of non-cardiac organs, such as the liver and adipose tissue (row 10 of *Table 1*).

## MMP-2 deficiency is associated with elevated secreted/circulating cardiac PLA2 activity

In 2015, a possibly new endocrine system was postulated, by which the heart influences cardio-hepatic lipid metabolism, hepatic sensitivity to dietary cholesterol, systemic inflammatory status, severity of fever and energy expenditure^[7^–^9]^. By investigating the pathophysiological consequences of MMP-2 deficiency in MMP-2 null (*Mmp2*^*-/-*^) mice, it was found that MMP-2 governs the secretion of a highly pro-inflammatory cardiac-specific phospholipase A2 activity (named ‘cardiac’ sPLA2). This finding provides a plausible and novel mechanism that could explain, at least partially, why human MMP-2 deficiency results in pediatric inflammatory arthritis with relentless bone loss, inflammation, cardiac developmental defects and other metabolic abnormalities such as hirsutism and dwarfism^[7^–^9]^. Two years after the identification of the MMP-2/cardiac sPLA2 axis^[7^–^9]^, there are key questions which warrant further investigation including: What is the molecular identity of the MMP-2-regulated sPLA2? What determines the cardiac origin of this sPLA2 in MMP-2 deficiency? We address these questions in the sections below.

## Cardiac sPLA2 may belong to the family of classical secreted phospholipases but its molecular composition is unknown

Up to now unsuccessful, previous attempts to identify cardiac sPLA2 have used targeted time-resolved immunofluorescence assays (TRIFA)^[8]^ or RT-PCR with reagents targeting the 31 different PLA2s (including classical and atypical, cytosolic and secreted enzymes)^[7^–^8]^ as well as conventional mass spectrometry, which is not inherently quantitative (the authors’ unpublished data). Activity inhibition studies have suggested that cardiac sPLA2 may be a mixture of indoxam-resistant (e.g., PLA2G1B, PLA2G2D, PLA2G2F, PLA2G10) and indoxam-sensitive (e.g., PLA2G2E, PLA2G5) sPLA2s or a new member of the sPLA2 family^[8]^.

To date, identifying the isoforms responsible for cardiac sPLA2 activity has been challenging calling for unbiased, highly sensitive and quantitative identification strategies such as a proteomics approach coupled with stable isotope-labelling with amino acids *in vivo* (mouse SILAC), a technique that has revolutionized the field of quantitative proteomics making it feasible to quantitate protein expression in mouse organs in two states^[52^–^53]^. Applying such a strategy (*Box 2*) has the added advantages of enabling the identification and quantification of all PLA2s deregulated (up- or down-regulated) in MMP-2-deficiency along with any other proteomics abnormalities. These resultant proteomic signature of MMP-2 deficiency could serve as biomarker of disease activity or as new therapeutic target in patients.

**Box 2:** On the molecular constituents of cardiac sPLA2

Since the first identification of its activity[^7^], the molecular identity of the MMP-2-regulated cardiac specific secreted PLA2 has remained elusive[^7^–^8^]. However, application of an unbiased quantitative proteomics approach could speed up the identification and enable quantitation of cardiac sPLA2 in biological samples. Proposed pillars of a quantitative proteomics approach to identify cardiac sPLA2 from *Mmp2*^*-/ -*^ mice include: (i) Use stable isotope labeling by amino acids (SILAC)[^52^–^55^] labeling integrated into the isolation strategy. (ii) BLAST-screen the identified cardiac sPLA2 against all known mouse and human PLA2s. (iii) Pursue a targeted "expression cloning strategy" where the cDNA of the identified enzyme is first expressed in, e.g., HEK-293, cells and next CCL7-induction of PLA2 activity is measured – the hypothesized pathway by which CCL7 elicits sPLA2 release[^8^] is depicted in *Box 3*.

## MMP-2, CCL7 and organ-homing immune cells govern cardiac sPLA2 release in an organ-specific fashion

Recent studies indicate that CCL7 (a small pro-inflammatory cytokine which is normally cleaved and inactivated by MMP-2) serves as stimulus for cardiac-specific release of sPLA2 activity^[8]^. This notion is supported by (a) *ex vivo* assays data^[7]^, showing that CCL7 stimulates sPLA2 release from cardiac, but not hepatic tissue and (b) the normalization of the cardio-hepatic lipid metabolic phenotype of MMP-2-deficient mice injected with neutralizing CCL7 antibodies, but not with isotype-matched non-immune IgG^[8]^. However, since CCL7 receptors are expressed on immune cells, cardiomyocytes and hepatocytes^[56^–^57]^, it is paradoxical that the liver of *Mmp2*^*-/-*^ mice does not exhibit elevated sPLA2 activity, whereas the heart of *Mmp2*^*-/-*^mice does, compared to wild-type mice. In *Box 3* we propose a mechanism that may clarify what makes cardiac sPLA2 "cardiac" in origin.

**Box 3:** On the mechanism that regulates the cardiac-specific origin of sPLA2

*Postulated heart/liver axis. *The red arrows trace the MMP-2-regulated CCL7-mediated pathway leading to the release of pro-inflammatory cytokines as well as secretion of cardiac sPLA2 and downstream lipid mediators (Lyso-phosphatidylcholine [PC], arachidonic acid [C20:4], prostaglandin E2 [PGE2]). Released into circulation, these mediators reach other organs where they influence the inflammatory status and lipid metabolism. Why sPLA2 is secreted from the heart and not the liver in MMP-2 deficiency? We postulate that the CD45^+^immune cells which reside in the liver of *Mmp2*^-/-^ mice express the CC-chemokine receptors (CCR) to which CCL7 binds[^56^–^57^]. These specific immune cells scavenge and consequently reduce the levels of free CCL7 below the threshold necessary for induction of sPLA2 by hepatocytes. This would not occur in the heart of *Mmp2*^-/-^ mice because of lack of significant immune cell homing[^8^] (as depicted in the enclosed Figure to the right, bottom). Published and unpublished observations by the authors support this notion including that: (i) CCL7 induces the transcription of classical sPLA2 isoenzymes as well as sPLA2 activity in Hepa-1c1c7 cells. (ii) CCR-expressing CD45^+^ immune cells are found homing to the liver (but not the heart) of *Mmp2*^-/-^ mice, compared to *Mmp2*^+/+^mice[^8^].	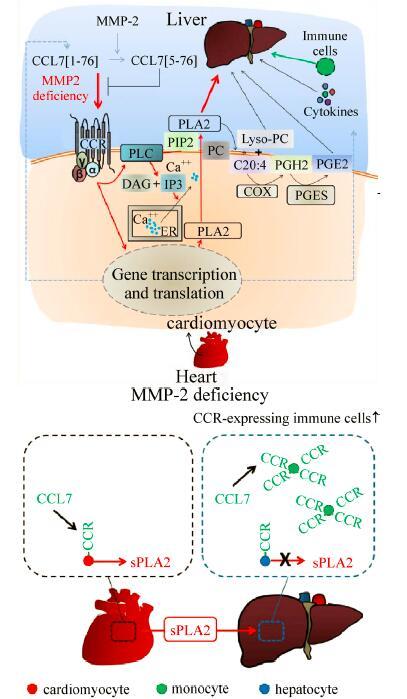

## Influence of the heart-centric MMP-2/CCL7/sPLA2 axis on lipid metabolism

A still-open question is whether MMP-2-mediated proteolysis of cytokines, such as CCL7, perturbs lipid metabolism *via* CCL7-receptor signaling pathways? To answer this question, *Box 4* describes two pathways by which the heart influences hepatic lipid metabolism and inflammation.

Future studies will provide precision to the first pathway described in *Box 4*, including the molecular identity (amino acid sequence) of the enzyme isoforms responsible for cardiac sPLA2 activity (*Box 2*) and deciphering the mechanism that regulates the cardiac-specific origin of sPLA2 in MMP-2 deficiency (*Box 3*).

**Box 4:** On the MMP-2 regulated CCL7-mediated modulation of lipid homeostasis

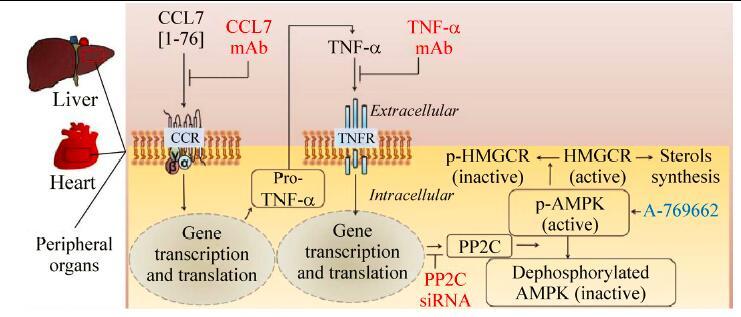 MMP-2 cleavage of CCL7 could influence lipid homeostasis through at least two pathways: Postulated pathway 1: Modulation of prostaglandin production by the CCL7 / cardiac sPLA2 axis[^7^–^8^]. (i) Cardiac sPLA2 activity, whose release is stimulated by CCL7, reaches non-cardiac organs, such as the liver, through the circulation; (ii) Cardiac sPLA2 hydrolyses membrane phospholipids in the liver releasing the fatty acid esterified at carbon-2 (typically, arachidonic acid, C20:4) and leaving behind a lysophospholipid (e.g., lyso-PC); (iii) Arachidonic acid is next converted into prostaglandin E2 by cyclooxygenases (as depicted in *Box 3*). Postulated pathway 2: Modulation of cholesterol metabolism by the CCL7 / tumor necrosis factor (TNF)-α /adenosine monophosphate-activated protein kinase (AMPK) axis. As illustrated, CCL7 can influence cholesterol homeostasis in target organs, including the heart and liver. Several key signaling events are: (i) CCL7 binds and activates CCR2[^56^]. (ii) A classical G-protein-coupled receptor-like calcium-dependent signaling cascade is elicited and pro-inflammatory genes, including TNF-α, are activated. (iii) Transcriptional induction of protein phosphatase 2C (PP2C) expression is stimulated; (iv) PPC2 reduces phosphorylation of active (phosphorylated) AMPK[^58^]; however, reduced AMPK phosphorylation can also be provoked by an interaction between AMPK and phosphoinositide 3-kinase enhancer A- a ubiquitously expressed GTPase and proto-oncogene – as recently reported[^59^]. (v) Metabolic enzymes including 3-hydroxy-3-methylglutaryl-coenzyme A reductase (HMGCR) are phosphorylated e.g., HMGCR in Ser 871) and inactivated. (vi) Mevalonate-the precursor of isoprenoids and sterols is synthesized. This pathway can be probed at various levels such as by antagonizing the actions of MCP-3/CCL7 and TNF-α with neutralizing monoclonal antibodies (mAb), by RNA interference to knockdown PP2C expression (PP2C siRNA) and by activating AMPK (A-769662).

## Conclusions

Some three decades ago, Dr. de Bold and colleagues identified endogenous peptide-hormones (ANP) which they found to stimulate a rapid and massive diuresis and natriuresis when injected in rats. Since this pioneering discovery, which demonstrated directly the endocrine function of the heart, there have been several new discoveries. These include but are not limited to: (i) the identification of other endogenous peptides (e.g., BNP, CNP) with natriuretic and vasodilator activity, (ii) the role of CNPs as hormones that can target various organs (e.g., the liver, brain, pancreas and intestine – not just the kidney) to influence metabolism, (iii) the role of the cardiac-specific miR-208a/MED13 axis to control whole body metabolism, (iv) a MMP-2/CCL7/sPLA2-mediated role played by the heart in inflammation and metabolism. These latter findings are potentially relevant for: (a) Conditions where MMP-2 activity is reduced by inactivating mutations (or polymorphisms) of MMP2 gene or medicinal drugs with MMP-inhibitory actions (although little is known about the prevalence of disorders caused by reduced MMP-2 activity) and (b) Disorders in which the expression of MMPs is deregulated- such as ischemic heart disease, arthritis, cancer, type 2 diabetes, obesity, hypercholesterolemia. Together, these discoveries could be vital for the diagnosis and for the design of new medicines for treating inflammatory and metabolic disorders.
